# Utilizing Computational Machine Learning Tools to Understand Immunogenic Breadth in the Context of a CD8 T-Cell Mediated HIV Response

**DOI:** 10.3389/fimmu.2021.609884

**Published:** 2021-02-18

**Authors:** Ed McGowan, Rachel Rosenthal, Andrew Fiore-Gartland, Gladys Macharia, Sheila Balinda, Anne Kapaata, Gisele Umviligihozo, Erick Muok, Jama Dalel, Claire L. Streatfield, Helen Coutinho, Dario Dilernia, Daniela C. Monaco, David Morrison, Ling Yue, Eric Hunter, Morten Nielsen, Jill Gilmour, Jonathan Hare

**Affiliations:** ^1^IAVI Human Immunology Laboratory, Imperial College, London, United Kingdom; ^2^Cancer Evolution and Genome Instability Laboratory, Francis Crick Institute, London, United Kingdom; ^3^Vaccine and Infectious Disease Division, Fred Hutchinson Cancer Research Center, Seattle, WA, United States; ^4^Medical Research Council/Uganda Virus Research Institute (MRC/UVRI) and London School of Health and Tropical Medicine (LSHTM), Uganda Research Unit, Entebbe, Uganda; ^5^Project San Francisco (PSF) Center for Family Health Research (CFHR), Kigali, Rwanda; ^6^Emory Vaccine Center, Emory University, Atlanta, GA, United States; ^7^Bitefirst, South Walsham, United Kingdom; ^8^Department of Health Technology, Technical University of Denmark, Lyngby, Denmark; ^9^IAVI, New York, NY, United States

**Keywords:** CD8 T-cells, HIV, T-cell epitopes, vaccines, machine learning

## Abstract

Predictive models are becoming more and more commonplace as tools for candidate antigen discovery to meet the challenges of enabling epitope mapping of cohorts with diverse HLA properties. Here we build on the concept of using two key parameters, diversity metric of the HLA profile of individuals within a population and consideration of sequence diversity in the context of an individual's CD8 T-cell immune repertoire to assess the HIV proteome for defined regions of immunogenicity. Using this approach, analysis of HLA adaptation and functional immunogenicity data enabled the identification of regions within the proteome that offer significant conservation, HLA recognition within a population, low prevalence of HLA adaptation and demonstrated immunogenicity. We believe this unique and novel approach to vaccine design as a supplement to vitro functional assays, offers a bespoke pipeline for expedited and rational CD8 T-cell vaccine design for HIV and potentially other pathogens with the potential for both global and local coverage.

## Introduction

Since the Human Immunodeficiency Virus (HIV) was first identified, 77.3 million people have become infected of which 35.4 million people subsequently died (1). Decades of research has enabled a comprehensive understanding of the structure, genetics, mechanism of infection, immune control and immune escape to emerge, resulting in novel targets for interventions, both as therapeutic targets, and for prophylaxis in the form of a broadly efficacious vaccine (2).

The structure of HIV lends itself to the development of vaccines that target the dominant surface glycoprotein gp120 and lead to the development of broadly neutralizing antibodies (3). Approaches to develop immunization regimes that will bias the development of this class of antibodies to provide prophylactic protection against HIV infection are under development with the first products entering clinical assessment (4). However, natural control of HIV viral load following the acute viral load burst is associated with a T-cell mediated response (5) and this suggests that a vaccine designed to raise T-cell responses may have efficacy if it is targeted to defined antigenic regions (6) including those with integral networked topology (7).

There are currently a number of T-cell vaccine candidates that utilize a variety of novel design approaches being tested in human clinical trials. The HIV Conserved vaccine (HIVCON) utilizes a conserved mosaic approach whereby regions of the proteome that have been identified as conserved within available databases are arranged in a specific regimen to both elicit T-cell responses to potential epitopes present within these regions, whilst limiting immunogenicity to the necessary joining or junctional regions (8). A second approach is to assemble known T-cell epitopes in a mosaic approach, whereby composite proteins are created to include common T-cells epitopes in a polyvalent design (9). A third approach, HIVACAT T-cell Immunogen, involves the construction a chimeric protein encoding 16 continuous segments of HIV derived from Gag, Pol, Vif, and Nef (10). There are pros and cons to all these approaches, but a potential caveat to utilizing conserved regions of the proteome is that historically pathogen diversity has been measured as the similarity or dissimilarity of sequences to each other, however a vaccine design should factor in how this pathogen sequence conservation is viewed by the host immune system.

Development and implementation of predictive models is becoming more commonplace as tools for candidate antigen discovery (11). This is highly relevant for HIV vaccine discovery where there is a staggering amount of complexity posed by diversity observed within individuals (12), within and between clades (13, 14) and within populations (15) making it a formidable challenge for rational T-cell vaccine design.

Here we present an *in silico* approach that complements the vaccine design strategies through the identification of HLA restricted antigenic regions within diverse HIV sequences based upon modeling of HLA restricted responses within individuals and linking these to disease progression via samples obtained from IAVI Protocol C, a longitudinal acute HIV infection study in east and sub-Saharan Africa covering multiple incident infection subtypes (16). We show that within a population, although HLA sequences show high levels of polymorphism, there are conserved, and over represented alleles associated with the >80% of the population covered within the study. In this study, we propose the use of the artificial neural network, NetMHCpan (17, 18) as a proxy to identify putative CD8 T-cell epitopes contained within the HIV transmitted founder virus (TFV) identified from the Protocol C clinical cohort of sub Saharan and East Africa. Using the transmitted founder virus sequence for relevant vaccine design is a well-established concept (19) and exploiting these predicted peptide/HLA interactions to generate additional novel metrics of HIV diversity adds another layer of information to facilitate vaccine design.

We believe that the size of the study cohort used in this investigation enables an extrapolation and scaling of the approach to global populations to enable a rationalized isolation and prediction of antigenic epitopes for any disease where a T-cell response is dominant in its control. By further informing vaccine strategies to focus the immune system against particular pathogens, incorporating potential immune recognition information into established models may increase the likelihood of success (20).

## Materials and Methods

### Cohort Characteristics

HLA profiles were evaluated from HIV+ volunteers enrolled in two IAVI-sponsored clinical cohorts. IAVI Protocol C is a prospective vaccine preparedness cohort studies of HIV-1 antibody negative heterosexuals or men who have sex with men in a Uganda Virus Research Institute/Medical Research Council/Wellcome Trust HIV-1 acquisition cohort study, and in a heterosexual sero-discordant couple's cohort study in Rwanda. Subjects were given HIV counseling, condom provision and regular HIV testing either monthly or quarterly. Those who seroconverted to HIV-1 were screened for stage of primary HIV-1 infection (16). IAVI Protocol G was a cross-sectional cohort of ~2,000 HIV positive individuals enrolled at 13 sites around the world in order to identify circulating broadly neutralizing antibodies (21).

### Near Full Length Transmitted Founder Genomes

The selection criteria for inclusion in the generation of near full length transmitted genomes is as previously described (22). For this analysis, 125 Near Full length transmitted Founder genomes were evaluated from across Africa (Table 1).

**Table 1:** Distribution of input transmitted founder proteome data.

**Clade**	***N***	**Distribution**
A	44	Kenya (19), Rwanda (18), Uganda (6), Zambia (1)
C	38	Kenya (2), Rwanda (1), Uganda (2). Zambia (33)
D	27	Kenya (3), Uganda (24)
Recombinant	16	Kenya (6), Rwanda (4), Uganda (8)

*Number of sequences from each country listed in parentheses*.

### HLA Distribution

The HLA binding predictor NetMHCpan was used to identify putative epitopes in 125 Transmitted Founder HIV-1 gag sequences derived from a cohort in Zambia (23). The distance between two sequences was defined as the percent of mismatched amino-acids in each 9 mer, summed across all 9 mers spanning the entire protein (i.e., a 500 aa protein contains 492 × 9 mers, each overlapping by 8 aa). This distance is dependent on sequences being aligned and therefore sequences sometimes contain gaps indicating insertions; this treats each gap character as an aa. Using this metric, the distance for the entire protein or for a subset of the 9 mers was determined; the epitope-based distance included only 9 mers in the alignment that were predicted to bind to at least one HLA allele. Binding was based on a threshold of 500 nM, though sensitivity analyses showed similar results with different thresholds.

#### Model Implementation

For each virus proteome a NetMHCpan simulation is performed for each of 46 Human Leukocyte Antigen (HLA) sequences. The 46 NetMHCpan result files for a virus proteome are then filtered to extract the peptide, HLA and rank binding where the rank binding is ≤ 2 [lower value is stronger binding (24, 25)]. This data is then loaded into a PostgreSQL database where an analysis tool is implemented in SQL stored procedures to identifies key peptides which appear in at least X viruses strains. The conservation metric X is defaulted to 2.2% of the total number of viruses initially being analyzed. The analysis tool then selects the virus that contributes the most of these key peptides. The selected virus and associated key peptides are then removed from the process and the next virus that contributes the most of the remaining key peptides is selected. The ranking process continues until all the key peptides are accounted for. The ranking results are then available to view or download at https://ibpt.iavi.org.

For comparison, set-building was performed a second time using randomly selected strains instead of choosing the strain that resulted in the greatest increase of peptide coverage.

#### HLA Adaptation Analysis

HLA adaptation analysis was performed as previously described (26). Briefly, each of the 319 peptides in the peptide set was aligned to the Zambian consensus sequence corresponding to the protein they were derived from and to HXB2. HLA adaptation was assessed using a list of statistically significant viral amino acid-HLA allele associations for Gag, Pol and Nef, previously described in Carlson et al. (27), as well as a new list generated for Rev, Tat, Vif and Vpr based on 295 sequences derived from chronically-infected individuals from Zambia plus 237 subtype C sequences downloaded from LANL (unpublished). A peptide was identified as adapted when the residue was positively correlated with the HLA or was any other residue other than the one negatively correlated with that HLA or the consensus (referred to as non-adapted). The correlation of residues to HLA was determined based on the number of HLA-linked polymorphisms relevant to the HLA alleles repertoire, as well as the number of polymorphisms located within well-defined CTL epitopes restricted by HLA alleles.

#### IFN-γ ELISPOT

The predicted peptides were evaluated for ability to induce T-cell responses by IFN-γ ELISPOT using bi-specific expanded CD8 T-cells as previously described (28). Briefly, PBMC were thawed and cultured in RPMI/10%FBS media supplemented with IL-2 (Sigma 50U/mL final concentration) and a CD3/CD4 bispecific antibody (Genscript) to expand CD8 T-cells. On Day 7 of expansion the CD8 population was assessed by Human IFN-γ 96 well ELISPOT (Mabtech) as per manufacturer's instructions. Peptide pools for 319 peptides were prepared as an 11 × 11 × 11 3D matrix with each peptide occurring in 3 unique pools. Positive responses were defined as the mean replicate count minus the mean background (mock) count where the mock controls must be <50 SFU/10^6^ PBMC and the media only wells <5 SFC/well).

#### Statistical Analysis

Statistical analyses were carried out using Prism version 6 (GraphPad Software, Inc., La Jolla, CA, USA). Python, Numpy and matplotlib were used to perform the Principal Component Analysis (PCoA).

The differences in sequence coverage determined by the different model parameters were assessed using Area Under Curve analysis and differences in the predicted coverage of each sequence was evaluated using a Kolmogorov-Smirnov test. For experimental ELISPOT data, normal distribution of data was assessed by the Shapiro–Wilk test. ELISPOT responses were compared by Mann-Whitney Test. Spearman correlation was used to assess relationships between ELISPOT responses and sequence priorities and coverage.

The data can be accessed through dataspace.iavi.org.

## Results

### HLA Distribution Within Specific Populations

**Figure 1:**
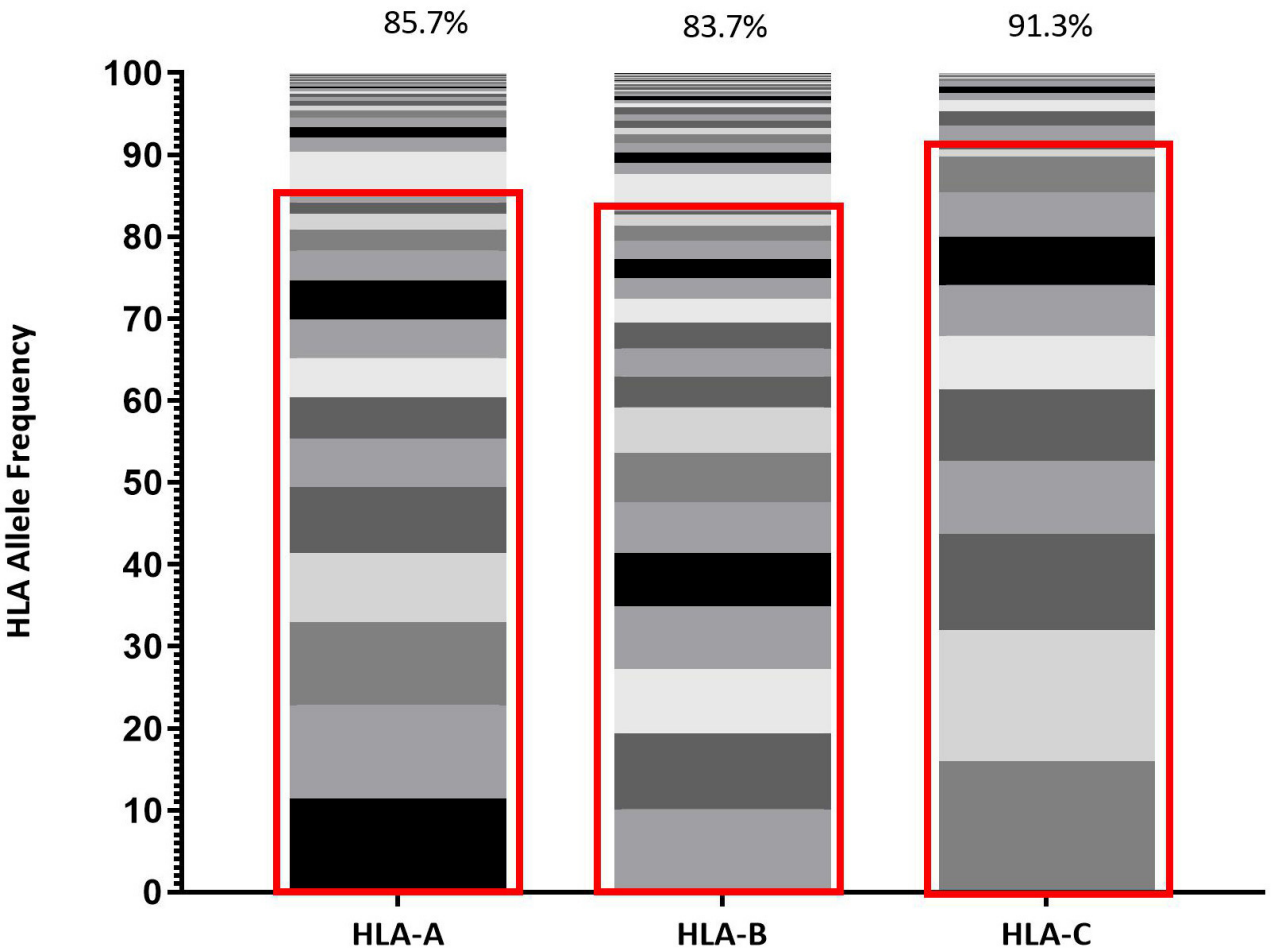
Frequency of each HLA Class I allele (HLA-A, HLA-B, and HLA-C) represented within IAVI Protocol C Alleles. Red boxes demarcate the allele frequencies contained within 13 pre-selected volunteers (Table 2) with percentage coverage listed above each stacked histogram plot. Seventeen Individual alleles contribute to HLA-A analysis, 21 Individual alleles contribute to HLA-B analysis, and 13 Individual alleles contribute to HLA-C analysis.

HLA distribution provides an important metric describing population diversity and correlates with the breadth of viable immune recognition within that population, which is relevant to both immune protection against pathogens and vaccine design strategies. Within Protocol C, all participants were screened for HLA composition upon enrollment and Figure 1 reflects the diversity of HLA Class I alleles within Protocol C (16) at a 2 field (4 digit) level of characterization (29).This data represents the HLA diversity of 613 participants and the prevalence of the HLA A, B, and C alleles is displayed as the relative percentage of the cohort.

Given the expected diversity of the HLA profile, it was an unexpected observation that >80% of the HLA Class I diversity of all alleles, are covered by 10 volunteers within the Protocol C cohort, supplemented with 3 individuals drawn from IAVI Protocol G (21) (Table 2). Furthermore, only an additional 11 Class I alleles with frequencies >1% but <5% within IAVI Protocol C are excluded from this analysis (Supplementary Table 1), indicating that even with a reduced subset of samples it may still be possible to capture the diversity of the full cohort HLA at the sequence level.

**Table 2:** Volunteers selected for determining HLA coverage within a population.

**Sample ID**	**HLA-A**	**HLA-A**	**HLA-B**	**HLA-B**	**HLA-C**	**HLA-C**
00C175058	A^*^02:05	A^*^23:01	B^*^07:05	B^*^49:01	C^*^07:01	C^*^07:02
00C191996	A^*^01:01	A^*^03:01	B^*^15:03	B^*^35:01	C^*^04:01	C^*^06:02
00C305154	A^*^68:02	A^*^74:01	B^*^15:03	B^*^18:01	C^*^02:10	C^*^05:01
00C362470	A^*^02:02	A^*^30:02	B^*^45:01	B^*^53:01	C^*^04:01	C^*^16:01
00C305125	A^*^23:01	A^*^34:02	B^*^08:01	B^*^15:10	C^*^07:01	C^*^08:02
00C191735	A^*^33:01	A^*^74:01	B^*^14:03	B^*^49:01	C^*^07:01	C^*^08:02
00C275031	A^*^23:01	A^*^30:02	B^*^07:02	B^*^15:10	C^*^03:04	C^*^07:02
00C275048	A^*^01:01	A^*^31:04	B^*^15:03	B^*^51:01	C^*^08:02	C^*^16:01
00C365005	A^*^29:02	A^*^30:02	B^*^42:01	B^*^57:03	C^*^17:01	C^*^18:01
00C365007	A^*^26:01	A^*^29:02	B^*^13:02	B^*^81:01	C^*^04:01	C^*^06:02
00G17616	A^*^02:01	A^*^66:01	B^*^53:01	B^*^58:02	C^*^04:01	C^*^06:02
00G27009	A^*^02:05	A^*^30:02	B^*^14:02	B^*^58:01	C^*^07:01	C^*^08:02
00G27188	A^*^02:05	A^*^30:01	B^*^07:02	B^*^27:03	C^*^02:02	C^*^07:02

**Figure 2:**
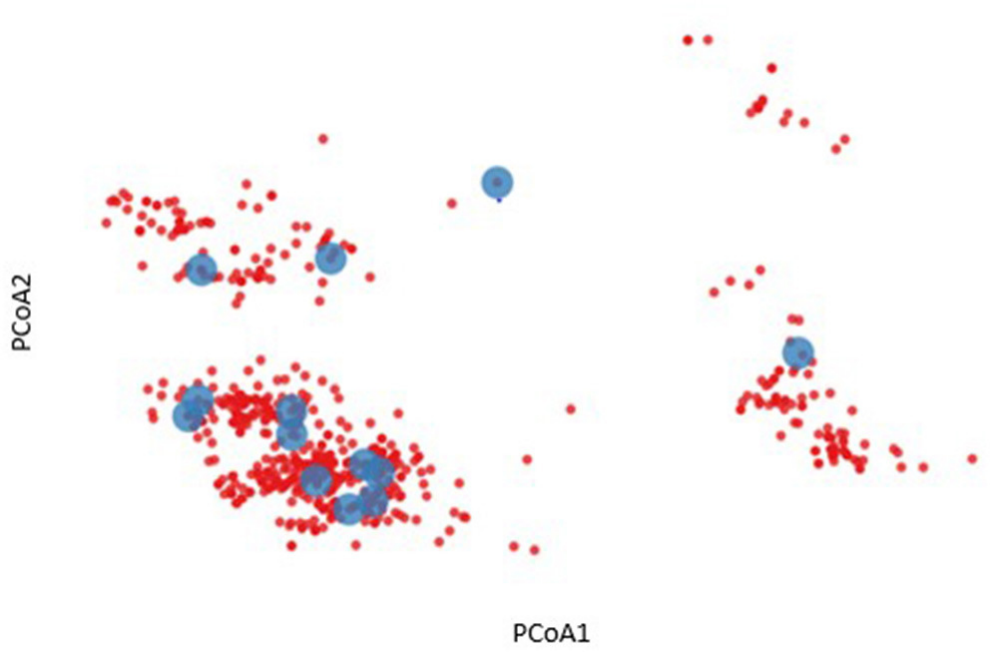
Two-dimensional representation of HLA diversity using Principal Component Analysis (PCoA). A HIV-1 Gag binding profile was predicted for every HLA allele using NetMHCpan and a set of transmitted founder sequences. The binding profile of each volunteer (red dot) was defined by taking the union of predicted binding for each of their HLA alleles. PCoA was performed using the pairwise similarity matrix of all volunteers, revealing distinct clusters of individuals. A subgroup of 13 volunteers were chosen to provide optimal coverage of the HLA binding profiles (blue dots).

To further characterize the diversity of the volunteers listed in Table 1, an HLA binding profile was modeled for each allele by predicting the binding affinity for each 9 mer peptide derived from a representative panel of HIV gag amino acid sequences using the NetMHCpan4.1 binding algorithm (18). This modeling enables us to define a binding profile of each HLA allele and each volunteer based on their HLA genotype. Based on the similarities of their binding profiles we were then able to cluster HLA alleles and/or volunteers to visualize and reassess HLA diversity (Figure 2). For example, a two-dimensional representation of HLA diversity in Protocol C can be generated using their pairwise HLA binding similarities and principal component analysis using a Spearman rank correlation-based distance such that alleles with higher positive correlation have a shorter distance while alleles with a lower correlation or negative correlation have a longer distance (D = [1—rho]/2). The analysis revealed distinct clusters of predicted HLA binding profiles (blue dots, Figure 2) which suggested that it was possible to identify a subgroup of Protocol C volunteers that were representative of the overall cohort HLA diversity (red dots, Figure 2).

**Figure 3:**
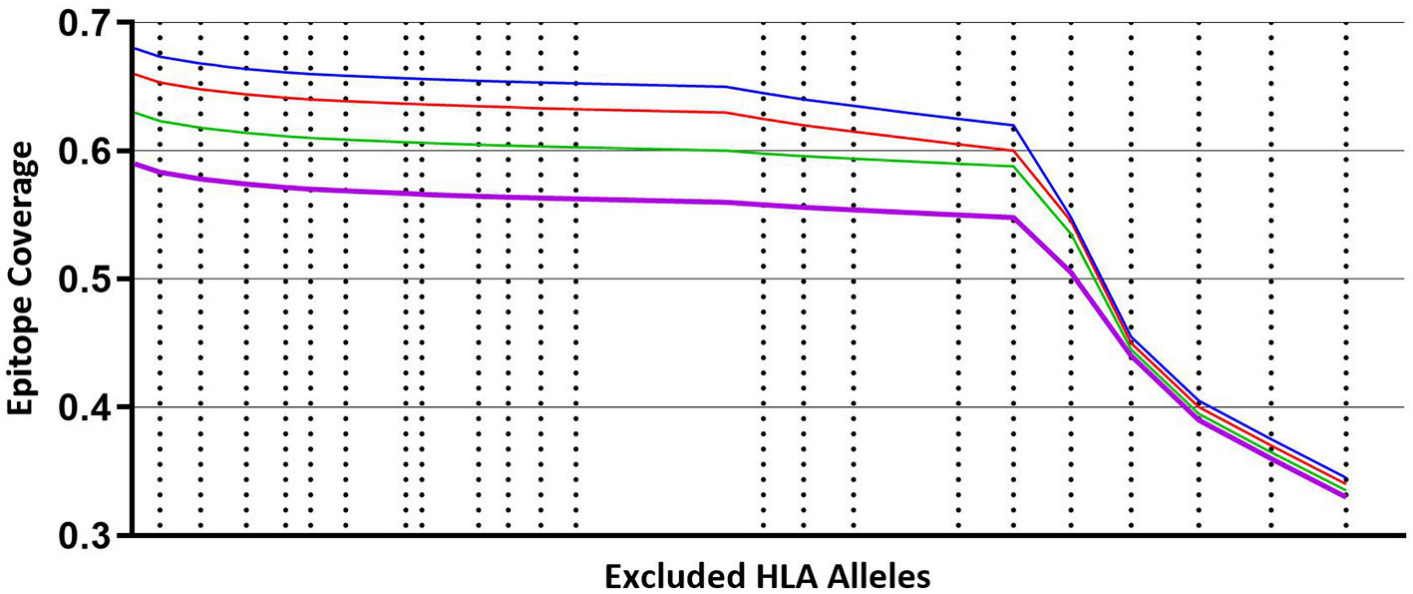
Coverage per predicted peptide calculated against a defined set of HLA alleles. Size of segments on X axis from left to right represents cumulative, combined HLA allele frequencies that are iteratively removed from the analysis, starting with least frequent alleles. Blue line—modeling using predicted 300 peptides. Red line—modeling using predicted 250 peptides. Green line—modeling using predicted 200 peptides. Purple line—modeling using predicted 150 peptides.

Figure 3 illustrates that coverage of the optimal peptide sets is influenced by the prevalence of HLA alleles within the prediction. As cumulative sets of HLA alleles are removed (starting with the least frequent alleles) there is minimal loss of epitope binding coverage observed (<10%) until a key inflection point is reached, leading to a precipitous loss of coverage, concordant with the frequency of the HLA alleles that are removed. Interestingly, the trend of minimal coverage loss at a minimal HLA frequency is observed independent of the size of the predicted peptide set with a comparable pattern observed for libraries of 300, 250, 200 and 150 peptides suggesting that while the HLA allele binding profile is peptide specific, it may also be independent of the peptides if a sufficient number are used.

### Development of a Predictive Model for HIV Diversity

Using NetMHCpan (at a 1% Binding Threshold), predicted 8, 9, and 10 mer epitopes were derived from TFV gag sequences (*N* = 125) obtained from HIV-infected volunteers enrolled in IAVI Protocol C, and identified in association with the HLA alleles present (listed in Table 1). Initial model development utilized a 1-select parameter where peptides were considered individually to determine the best coverage. This resulted in the prediction of 6,562 peptides (Supplementary Table 2) and no difference in best coverage mapping vs. random selection by Kolmogorov-Smirnov test (*p* = 0.4670) was observed. Subsequent analysis of this model revealed that 4,812 (73%) of these peptides were either unique to an individual gag sequence or present in only two gag sequences. If only peptides that were present in ≥3 virus sequences (3-select best) were considered, this led to the prediction of 1,750 peptides (26.7% of the 1-select best model), which was shown to be more effective at mapping coverage than randomly selecting peptides (*p* < 0.0001) (Supplementary Figure 2, Supplementary Table 2).

Further model development evaluated the effect of varying the binding threshold on the predicted outcomes. The binding threshold is a measurement of confidence that a predicted peptide will associate with the prescribed HLA, for example a 1% binding threshold factors in a 1% false positive rate. Running the model whilst varying binding thresholds at 0.5, 1, and 2% resulted in the identification of 955, 1,750 and 3,023 peptides, respectively (Supplementary Table 2). No difference was observed in coverage when the 1% binding threshold was set to a less stringent 2% or a more stringent 0.5% (*p* = 1 *and p* = 0.6430), therefore a 1% binding threshold was selected for all future analyses to maximize coverage whilst being able to distinguish additional conserved epitopes (Supplementary Figure 2).

### Modeling of HIV Diversity for Full Length Transmitted Founder Proteomes

**Figure 4:**
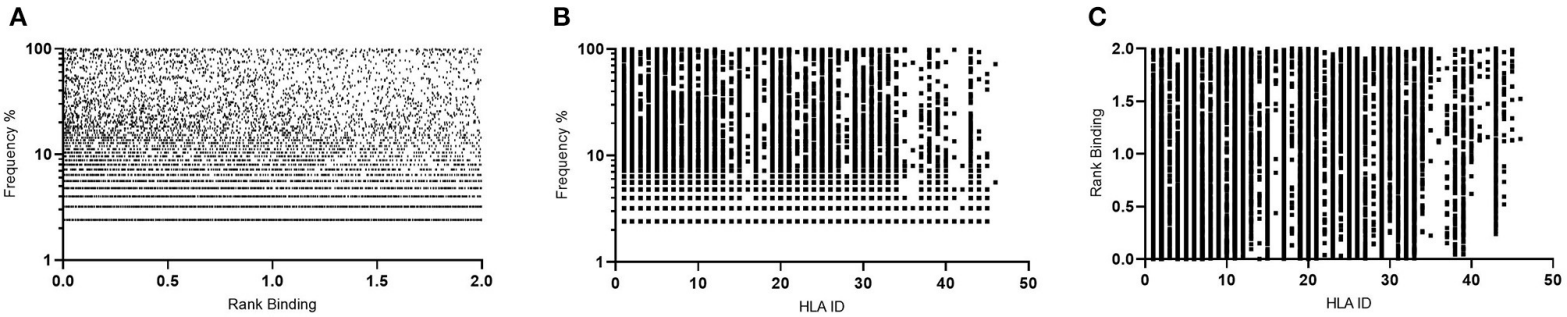
Affinity plots for all predicted peptides with conservation of ≥2.2% (*n* = 14,953). **(A)**—Predicted peptide affinity (Rank Binding) vs. primary associated HLA. **(B)**—Predicted peptide affinity (Rank Binding) vs. peptide frequency within transmitted founder proteome. **(C)**—Predicted peptide frequency vs. primary associated HLA.

These same parameters (1% Binding Threshold, Rank Binding ≤2, Peptide Conservation ≥2.2%) were then applied to analyze 125 Transmitted Founder proteome sequences (excluding envelope) derived from IAVI's Protocol C (see Tables 1, 3 for input sample data and model parameters). The initial evaluation identified 14,953 predicted peptides occurring with a frequency of 2.2% in our population. This peptide set covers all predicted affinities and coverages and may represent multiple HLA interactions/peptide. To evaluate the distribution of affinities to the primary associated HLAs with Rank Binding scores were assessed (Figure 4A). Rank binding is an alternative metric for HLA:peptide affinity that can be deployed in order to normalize the large diversity in the range of predicted binding values for the different HLA molecules and therefore limit bias derived from over-represented HLA (18). Rank binding assigns each peptide a score with peptides annotated as a strong binder if their score is <0.5 or a weak binder if the score is 0.5–2.0.

**Table 3:** Model parameters.

**Parameter**	**Values**
Binding threshold	1%
HLA allele contributions	All HLA alleles from 13 individuals (Table 1)
HLA haplotype weighting	0
Rank binding	<2.0
Peptide conservation (%)	2.2
Peptide length	8, 9, 10, and 11 mers

To further control for potential bias within the peptide-HLA interactions, the peptides were then analyzed by both affinity and Rank Binding to all predicted HLA interactions (Figure 4B) and the frequency that these peptides occurred in the population in the context of the specific HLA alleles (Figure 4C).

This analysis identified a range of predicted binding profiles for the different peptide-HLA interactions (see Supplementary Table 1 for full HLA allele identities). HLA-A*02:02, HLA-A*31:04, and HLA-B*15:03 were identified as having particularly high predicted affinity peptide interactions, whereas HLA-B*14:03, HLA-B*15:10, and HLA-C*04:01 have much lower predicted affinity peptide interactions. This differential pattern of binding may be explained due to the large diversity in the range of predicted binding values for the different HLA molecules. When plotted using the Rank Binding metric these differences are less pronounced although trends of stronger associations to specific HLA alleles remain.

Implementing these frequency and binding thresholds to identify HIV-specific predicted CD8 T-cell epitope peptides can be used as a functional metric to assess HIV diversity. By assuming that these predicted peptides provide a novel tool for ranking HIV proteome diversity, it is possible to assign a coverage gain value to each sequence and then utilize those values to rank each sequence for the coverage it provides within the sample population. By implementing these calculations, it is then possible to identify the sequences that are necessary to obtain the optimum level of epitope restricted sequence coverage.

**Figure 5:**
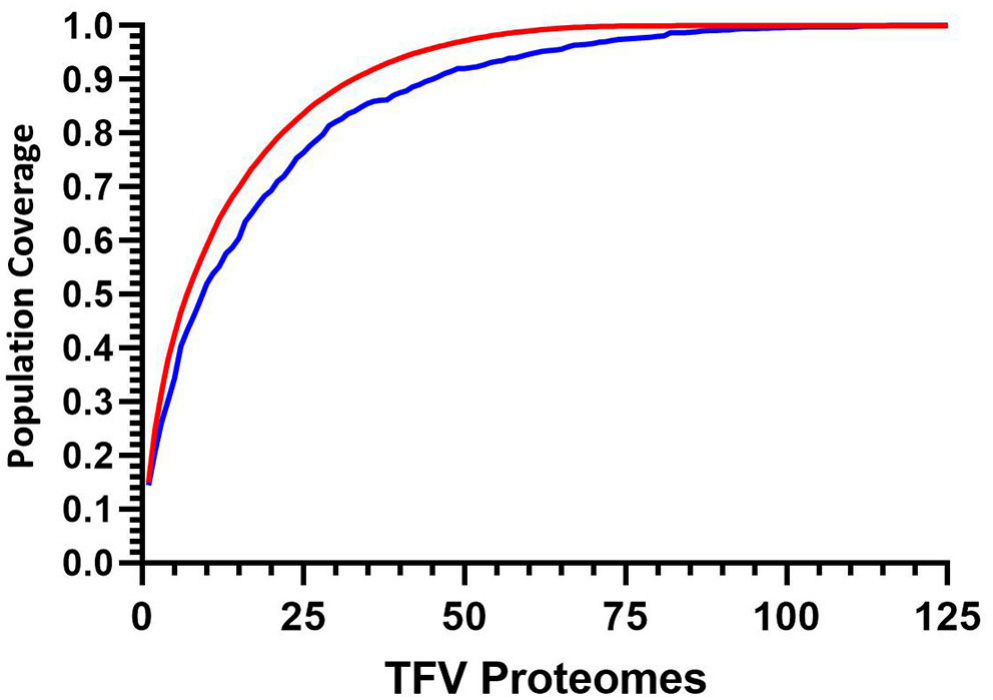
Cumulative coverage distribution plots of full length transmitted founder gag sequences using a 3-select coverage model and a 1% Binding Threshold, 3-Select best (red), and 3-Select random (blue). *P-*values calculated using Kolmogorov-Smirnov test.

The implementation of this model can then be used to target and prioritize individual proteomes. Figure 5 illustrates how for 125 transmitted founder virus proteomes, achieving 90% coverage requires 33 prioritized viruses, which decreases to 22 and 16 viruses if 80 or 70% coverage is desired, respectively (data not shown). Importantly, ~40% more viruses are required to achieve 90% coverage if sequences are randomly selected (*n* = 45 *p* < 0.0001).

### *In silico* Characterization of Predicted Peptides

Whilst evaluating peptides at a prevalence of ≥2.2% is desirable from the perspective of understanding population coverage, it is more challenging to map potential regions of the proteome for anti-HIV T-cell specificities due to the large levels of redundancy and overlap in evaluating each HLA/epitope interaction. By selecting HIV sequence coverage as the primary parameter and predicted affinity as a secondary characteristic the peptide library should contain both predicted high and lower affinity epitopes with optimum coverage, that may have functionality if represented at high enough abundance. Through further stratifications of the predicted peptide set to limit sequence overlap, and through assigning a minimum population coverage of 40% (selected to maintain sequence conservation and not introduce multiple sequence variations) resulted in the identification of 957 peptides. Of these peptides, an unbiased subset of 319 peptides were selected at random from across the proteome for further *in silico* and *in vitro* characterization.

HLA adaptation in a particular epitope is defined as the presence of a particular residue that has been statistically linked to an individual HLA, indicating a process of immune selection in that context (26). Vaccine design utilizing conserved epitopes may unwittingly overlook the observation that not all epitopes in the transmitted virus will be consensus and in fact, some may actively promote CTL escape (30). The peptides identified by the 3-select model were evaluated for predicted HLA adaptation as previously described (26). Of these peptides 75/319 were identified as containing a residue that was adapted, although interestingly the predicted adaptation was against alternative HLA alleles not predicted by the model for 70/75 predicted peptides with only 2 out of 5 adapted peptides associating to the primary HLA allele (Supplementary Table 4).

### Predicted Peptide *in vitro* Characterization

To confirm that the selected subset of predicted peptides were recognized by anti-HIV specific T-cells, IFNγ ELISPOT assays were performed using a 3D Matrix approach described elsewhere (31). The peptides were evaluated in samples from 23 HIV+ volunteers at a single time point ~12 months post-estimated date of infection to determine the contribution of individual HLA and input sequences and correlate these metrics to observed T-cell responses. These volunteers were identified for whether their transmitted founder sequences were included (Group “Seq In”-−10 volunteers) or excluded in the modeling analysis (Group “Seq Out” 13 volunteers). ELISPOT responses were also evaluated at a second time point ~60 months post EDI, although this data was not included in the analysis, Supplementary Table 5).

**Figure 6:**
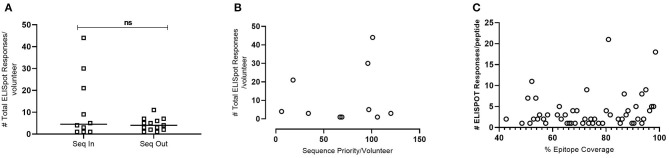
IFNγ ELISpot responses observed in HIV+ Volunteers. **(A)**—Number of total ELISpot responses observed in volunteers whose transmitted founder proteome sequence was included within the *in-silico* prediction (Seq In: *N* = 10) and volunteers whose transmitted founder proteome sequence was not included within the *in-silico* prediction (Seq Out: *N* = 13). Shapiro-Wilk values Seq In: *W* = 0.7887, *p* = 0.0008. Seq Out: *W* = 0.8976, *p* = 0.0315. Mann-Whitney test, *p* = 0.6215. **(B)**—Correlation of total number of ELISpot responses in volunteers whose transmitted founder proteome sequence was included within the *in-silico* prediction against the order of priority the sequence was predicted to occur (Spearman Correlation; *r* = 0.1356, *p* = 0.2209). **(C)**—Correlation of total number of ELISpot responses in volunteers whose transmitted founder proteome sequence was included within *in silico* prediction against the % coverage each epitope represented (Spearman Correlation; *r* = 0.2695, *p* = 0.0357).

To evaluate whether the model introduced bias from volunteers who contributed their transmitted founder sequence compared to volunteers whose sequence was not included, IFNγ ELISpot responses were analyzed at 12 months post-estimated date of infection. The results indicated no significant difference in the median number of responses per volunteer (median responses/volunteer n = 4.5 group Seq In vs. median responses/volunteer n = 4 group Seq Out, Figure 6A). Further analysis revealed that there was no bias in responses toward the volunteers with sequences predicted to contribute the most coverage vs. those volunteers whose sequences contributes less to coverage (Figure 6B). Assessing the number of individual ELISPOT responses per peptide revealed a trend toward increasing number of responses as the conservation of the peptides increases, although this correlation was not significant (Figure 6C).

## Discussion

We propose that through the addition to the predictive algorithm NetMHCpan, two novel parameters are defined that can be exploited to aid the rational selection of T cell vaccine immunogens. The first parameter confers the ability to assign a diversity metric to the HLA profile of individuals within a population. The existing metrics of 2-field characterization of HLA alleles enables frequencies of alleles to be calculated but has several limitations when considering HLA diversity/similarity. A clear limitation is that the peptide binding profile of two alleles may not be strongly associated with the similarity of their 2-field allele representation (32). A second method for characterizing HLA allele diversity involves the assessment of the amino acid sequence of the MHC protein with a focus on the peptide binding groove (33). Building on this idea, an alternative, advantageous approach to assessment of the diversity of the HLA frequency may therefore be to use computationally predicted peptide binding of the HLA alleles based on machine learning algorithms trained on functional binding data as well as the amino acid sequences of the HLA proteins (17). We propose an alternative metric of HLA diversity that utilizes the predicted binding affinity of a reference amino acid sequence to assign each HLA allele an individual binding score. By evaluating the individual HLA profiles of individuals in a studied cohort, it is then possible to calculate a combined HLA diversity metric. Using these values, individual volunteers can be mapped within specific populations and distance scores calculated between each allele and each volunteer. Using this approach, we have demonstrated that it is possible to select individuals within a cohort that are “representative” of the population from which they are drawn. Implementing this stratification of volunteers may have implications for the design of smaller experimental clinical trials.

The second parameter is a metric for HIV diversity determined through the perspective of predicted binding of putative CD8 T-cell/HLA epitopes. Previous evaluations of HIV diversity rely on sequence clustering and alignments to order individual sequences. This alignment is appropriate for comparing the actual sequence of a virus genome or proteome, however this approach is limited for evaluating how an individual may recognize a specific proteome. By considering sequence diversity in the context of an individual's HLA profile and therefore potential CD8 T-cell immune repertoire, an additional diversity metric can be layered to represent how an individual may be predicted to view a virus proteome and through combining the *in-silico* metrics, it is possible to rank HIV proteome sequences by the coverage they provide within the population across individuals. This ability to rank sequences according to putative immunogenic breadth additionally enables the interpretation of functional immunological killing assays like the viral inhibition assay (34, 35). Traditionally these assays have been interpreted as a binary assessment of the number of viruses inhibited. Using these novel metrics, it would now be possible to assign a population coverage score to each virus or panel of viruses and as such be able to provide an estimate as to the potential anti-virus killing activity of a volunteer based on the pattern of viruses they can inhibit.

IFNγ ELISpot analysis using the peptides predicted by the model revealed that there was no significant increase in the number of ELISpot responses/volunteer if the individual's TFV proteome sequence was included in the prediction compared to the number of responses/volunteer if an individual's TFV proteome was not included. This data indicates that using a subset of samples for prediction has not created any inward bias toward the input source but is representative of the population. The frequency of responses observed in this study for both groups are lower than those previously reported (36–38), however this reflects the increased stringency incorporated into the development of this peptide set whereby only peptides with a predicted coverage > 40% were included. By way of comparison, the conservation threshold for the peptides evaluated by Kunwar et al. (36) and Sunshine et al. (38) were 15 and 5%, respectively, with a response rate/volunteer of 7 and 12 epitopes, respectively.

This hypothesis indicates that through understanding the conservation, adaptation and functional score assigned to any population of target sequences, it is possible to embed this metric within algorithms to fully evaluate potential immunogenicity within the context of sequence conservation and HLA allele frequency and may contribute to expedited vaccine design and iterative testing strategies aimed at inducing protective CD8 mediated T-cell immunity. The principals underpinning this approach have applicability to other disease models and geographies for which comparative input data is available and protective CD8 responses are desirable.

## Data Availability Statement

The datasets presented in this study can be found in online repositories. The names of the repository/repositories and accession number(s) can be found in the article/Supplementary Material.

## Ethics Statement

The studies involving human participants were reviewed and approved by the local ethics review boards, including the Kenya Medical Research Institute Ethical Review Committee, the Kenyatta National Hospital Ethical Review Committee of the University of Nairobi, the Rwanda National Ethics Committee, the Uganda Virus Research Institute Science and Ethics Committee (Currently the UVRI Research Ethics Committee) and the Uganda National Council of Science and Technology, the University of Cape Town Health Science Research and Ethics Committee, the Bio-Medical Research Ethics Committee at the University of KwaZulu Natal, the University of Zambia Research Ethics Committee, and the Emory University Institutional Review Board. Written informed consent was obtained for all participants. The patients/participants provided their written informed consent to participate in this study.

## Author Contributions

EMc and JH wrote the manuscript, provided conceptual input, and data analysis. AF-G, RR, DM, DCM, LY, and MN provided technical expertise and contributed to manuscript. JD, HC, and CS performed ELISPOT assays. DD, SB, AK, GU, GM, and EMu were integral to providing the input viral sequence data. EH and JG provided key supervision and support. All authors contributed to the article and approved the submitted version.

## Conflict of Interest

The authors declare that the research was conducted in the absence of any commercial or financial relationships that could be construed as a potential conflict of interest.
