# Supplementary material for: Utilizing Computational Machine Learning Tools to Understand Immunogenic Breadth in the Context of a CD8 T-Cell Mediated HIV Response
Source: Front Immunol. 2021 Feb 18;12:609884. doi: 10.3389/fimmu.2021.609884 (PMC7930081; doi:10.3389/fimmu.2021.609884)
Supplement: Supplementary file 1 [file Data_Sheet_1.docx]

**Supplementary Tables & Figures**

Supplementary Figure 1 Comparison of 3-select and 1-select models. Cumulative coverage distribution plots of transmitted founder gag sequences using either a 3-select coverage model (3-Select best – blue; 3-Select Random – red) or a 1-select coverage model (1-Select best – green; 1-Select Random –purple)

Supplementary Figure 2. Cumulative coverage distribution plots of transmitted founder gag sequences using a 3-select coverage model and varying the binding threshold. 2% Binding Threshold, 3-Select best (blue), 2% Binding Threshold, 3-Select random (red), 1% Binding Threshold, 3-Select best (green), 1% Binding Threshold, 3-Select random (purple), 0.5% Binding Threshold, 3-Select best (orange), 0.5% Binding Threshold, 3-Select random (black)

| HLA-A | HLA-A Frequency | HLA-B | HLA-B Frequency | HLA-C | HLA-C Frequency |
| --- | --- | --- | --- | --- | --- |
| **A*02:01 (2)** | **11.40%** | **B*53:01 (32)** | **10.08%** | **C*06:02 (40)** | **15.98%** |
| **A*68:02 (15)** | **11.40%** | **B*15:03 (23)** | **9.34%** | **C*04:01 (39)** | **15.98%** |
| **A*30:02 (10)** | **10.17%** | **B*45:01 (29)** | **7.85%** | **C*07:01 (41)** | **11.75%** |
| **A*30:01 (9)** | **8.43%** | **B*58:02 (35)** | **7.60%** | **C*17:01 (45)** | **8.86%** |
| **A*23:01 (6)** | **8.02%** | **B*42:01 (28)** | **6.53%** | **C*02:10 (37)** | **8.86%** |
| **A*74:01 (16)** | **5.95%** | **B*07:02 (17)** | **6.20%** | **C*07:02 (42)** | **6.46%** |
| **A*29:02 (8)** | **5.04%** | **B*58:01 (34)** | **5.95%** | **C*16:01 (44)** | **6.21%** |
| **A*01:01 (1)** | **4.79%** | **B*15:10 (24)** | **5.62%** | **C*08:02 (43)** | **5.88%** |
| **A*02:02 (3)** | **4.71%** | *B*44:03* | *3.97%* | **C*03:04 (38)** | **5.46%** |
| **A*03:01 (5)** | **4.71%** | **B*49:01 (30)** | **3.80%** | **C*18:01 (46)** | **4.30%** |
| *A*36:01* | *4.63%* | **B*57:03 (33)** | **3.39%** | *C*07:04* | *2.24%* |
| **A*34:02 (13)** | **3.64%** | **B*18:01 (25)** | **3.14%** | *C*03:02* | *1.74%* |
| **A*66:01 (14)** | **2.64%** | **B*14:02 (21)** | **2.89%** | *C*04:07* | *1.32%* |
| **A*02:05 (4)** | **1.90%** | **B*35:01 (27)** | **2.56%** | **C*02:02** | **0.91%** |
| *A*68:01* | *1.82%* | **B*81:01 (36)** | **2.31%** | C*14:02 | 0.91% |
| **A*33:01 (12)** | **1.40%** | **B*08:01 (19)** | **2.23%** | C*15:02 | 0.83% |
| *A*30:04* | *1.24%* | **B*51:01 (31)** | **1.90%** | C*12:03 | 0.66% |
| *A*24:02* | *1.16%* | **B*13:02 (20)** | **1.32%** | **C*05:01** | **0.66%** |
| **A*26:01 (7)** | 1.16% | *B*14:01* | *1.32%* | C*16:02 | 0.25% |
| A*33:03 | 0.83% | *B*44:15* | *1.24%* | C*01:02 | 0.17% |
| A*01:03 | 0.58% | *B*57:02* | *1.24%* | C*03:03 | 0.17% |
| A*32:01 | 0.58% | B*15:17 | 0.99% | C*06:03 | 0.08% |
| A*80:01 | 0.50% | B*41:01 | 0.83% | C*15:16 | 0.08% |
| A*01:02 | 0.41% | B*42:02 | 0.83% | C*08:04 | 0.08% |
| A*30:09 | 0.33% | B*39:10 | 0.83% | C*12:02 | 0.08% |
| A*31:01 | 0.33% | B*15:16 | 0.83% | C*16:04 | 0.08% |
| **A*31:04 (11)** | **0.33%** | B*40:16 | 0.50% |  |  |
| A*02:14 | 0.25% | **B*27:03 (26)** | **0.41%** |  |  |
| A*43:01 | 0.25% | **B*07:05 (18)** | **0.41%** |  |  |
| A*66:02 | 0.25% | B*37:01 | 0.41% |  |  |
| A*02:04 | 0.17% | B*73:01 | 0.41% |  |  |
| A*11:01 | 0.17% | B*15:31 | 0.33% |  |  |
| A*26:12 | 0.17% | B*47:01 | 0.25% |  |  |
| A*23:02 | 0.08% | B*15:01 | 0.25% |  |  |
| A*26:03 | 0.08% | B*41:02 | 0.25% |  |  |
| A*29:01 | 0.08% | B*35:02 | 0.25% |  |  |
| A*31:03 | 0.08% | B*18:03 | 0.17% |  |  |
| A*66:03 | 0.08% | B*47:03 | 0.17% |  |  |
| A*74:02 | 0.08% | B*40:12 | 0.17% |  |  |
| A*74:03 | 0.08% | **B*14:03 (22)** | **0.17%** |  |  |
| A*74:05 | 0.08% | B*50:01 | 0.17% |  |  |
|  |  | B*82:02 | 0.17% |  |  |
|  |  | B*27:05 | 0.08% |  |  |
|  |  | B*07:51 | 0.08% |  |  |
|  |  | B*52:01 | 0.08% |  |  |
|  |  | B*15:47 | 0.08% |  |  |
|  |  | B*15:37 | 0.08% |  |  |
|  |  | B*15:83 | 0.08% |  |  |
|  |  | B*35:25 | 0.08% |  |  |
|  |  | B*56:01 | 0.08% |  |  |
|  |  | B*57:01 | 0.08% |  |  |

Supplementary Table 1. HLA allele frequency within Protocol C. Alleles in bold are represented within 13 pre-selected volunteers. Alleles in italics are alleles with a population frequency of >1% and <5% that are excluded from the analysis. Numbers in bold italic parentheses correspond to primary associated HLA allele identification in Figure 4

|  | # Peptides | AUC | | X=1, y= | | P Value |
| --- | --- | --- | --- | --- | --- | --- |
|  |  | Best | Random | Best | Random |  |
| 1-select | 6562 | 101 | 95.05 | 120 | 120 | 0.4670 |
| 3-select | 1750 | 111.5 | 108.3 | 51 | 91 | <0.0001 |
| 2% Binding | 3023 | 112.7 | 108.1 | 63 | 98 | <0.0001 |
| 1% Binding | 1720 | 113.4 | 108.5 | 57 | 98 | <0.0001 |
| 0.5% Binding | 955 | 114.2 | 108.1 | 50 | 98 | <0.0001 |

Supplementary Table 2. Model development comparing peptide conservation with levels of predicted binding affinities. P value calculations were by Kolmogorov-Smirnov test.

| KP715746 | KF716477 | KF716504 | KP715844 | KP715777 |
| --- | --- | --- | --- | --- |
| KF716481 | MT942773 | KP715798 | KP109491 | KF716501 |
| MT942832 | KR820323 | MW006079 | MT942867 | KF716470 |
| KR820421 | KP715769 | MW006054 | MT942955 | MT942819 |
| KF716478 | MW006067 | KC596071 | MT942776 | KP715843 |
| MW006071 | KP109490 | MT194610 | KP109493 | MT942790 |
| KP715751 | MW006064 | KP174771 | KF716466 | KP109495 |
| MW006069 | KP109494 | MT347679 | KP715790 | MW006059 |
| MT942927 | KF716489 | KF716490 | KF716469 | KP109492 |
| MT942805 | MT942755 | MT027070 | MT942928 | KF716473 |
| MW006075 | KP715734 | KU749427 | KF716488 | KF716479 |
| KP715791 | MT942748 | KU749429 | KF716467 | KF716476 |
| KF716503 | MT942896 | KP109496 | KF716487 | KJ190262 |
| MT942880 | KP109497 | KP715832 | MT942899 | KU749428 |
| KP715771 | KF716482 | MW006062 | MW006070 | KF716475 |
| KU749430 | KF716483 | MT942941 | MW006065 | MT942836 |
| KF716471 | MT195144 | KF716486 | MT942708 | MT194178 |
| KF716480 | MT942722 | KF716484 | KF859747 | KP715741 |
| KC596072 | KU749425 | KF716472 | MT195515 | KR820393 |
| MT194327 | KF859745 | KR820366 | KF716468 | MW006075 |
| MW006068 |  |  |  |  |

Supplementary Table 3. Genbank Accession numbers of input transmitted founder virus sequences

| Predicted Peptide | Primary HLA | Adapted HLA | Match | Predicted Peptide | Primary HLA | Adapted HLA | Match |
| --- | --- | --- | --- | --- | --- | --- | --- |
| AAEWDR**L**HPV | B*49:01 | A*30 | N | **A**ETFYVDGAA | A*68:02 | B*53:01 | N |
| AEWDR**L**HPV | B*45:01 | A*30 | \|N | G**A**ETFYVDGA | B*45:01 | B*53:01 | \|N |
| MR**E**PRGSDI | C*07:01 | C*03:02 | N | TFYVDGAA**N**R | A*31:04 | B*15:10 | N |
| IYK**R**WIILGL | A*23:01 | A*30 | N | FYVDGAA**N**R | A*33:01 | B*15:10 | N |
| YK**R**WIILGL | B*14:03 | A*30 | N | RETK**L**GKAGY | A*30:02 | A*68 | N** |
| **R**WIILGLNKI | A*23:01 | A*30 | N | ETK**L**GKAGYV | A*68:02 | A*68 | Y** |
| RPEP**T**APPA | B*07:02 | A*30:04 | N | YVTDRGRQ**K** | A*34:02 | C*07:01 | N |
| **E**VGFPVRPQV | A*68:02 | A*03:01/B*44 | N | VTDRGRQ**K**V | C*05:01 | C*07:01 | N |
| E**V**GFPVRPQV | B*07:02 | C*02:02 | N | VTDRGRQK**V** | C*05:01 | B*57:02 | N |
| EVGFPV**R**PQV | B*07:02 | B*44/C*08 | N | DKLVS**S**GIRK | A*03:01 | A*74 | N |
| GFPV**R**PQVPL | B*07:02 | B*44/C*08 | N | KLVS**S**GIRK | A*03:01 | A*74 | Y |
| FPV**R**PQVPL | B*07:02 | B*44/C*08 | N | **S**GIRKVLFL | B*08:01 | A*74 | N |
| FPV**R**PQVPLR | B*07:02 | B*44/C*08 | N | WRAMAS**D**FNL | B*14:03 | B*49:01 | N |
| V**R**PQVPLRPM | B*07:02 | B*44/C*08 | N | RAMAS**D**FNL | C*03:04 | B*49:01 | N |
| RQ**E**ILDLWVY | A*30:02 | B*57:02/B*57:03 | N | CTHLEGK**V**IL | B*15:10 | B*15:10 | Y |
| RQ**E**ILDLWV | B*49:01 | B*57:02/B*57:03 | \|N | THLEGK**V**IL | B*14:03 | B*15:10 | Y* |
| Q**E**ILDLWVY | B*18:01 | B*57:02/B*57:03 | N | HLEGK**V**ILVA | B*45:01 | B*15:10 | N |
| **K**EALLDTGA | B*07:02 | B*35 | N | LEGK**V**ILVA | B*45:01 | B*15:10 | N |
| LPGKWKP**K**M | B*07:02 | B*15:03 | N | LEGK**V**ILVAV | B*49:01 | B*15:10 | N |
| KP**K**MIGGIG | A*30:02 | B*15:03 | N | VHTDNG**S**NF | B*15:10 | C*02 | N |
| **K**MIGGIGGF | A*02:02 | B*15:03 | Y* | HTDNG**S**NFT | A*01:01 | C*02 | N |
| **K**MIGGIGGFI | B*07:02 | B*15:03 | N | FTSA**A**VKAA | A*68:02 | A*74 | N |
| PAIFQSSM**T**K | A*03:01 | C*16 | N | SA**A**VKAACW | B*53:01 | A*74 | N |
| NTPIFAI**K**K | A*34:02 | A*30 | N | SA**A**VKAACWW | B*53:01 | A*74 | N |
| NTPIFAI**K**KK | A*34:02 | A*30 | N | A**A**VKAACWW | B*53:01 | A*74 | N |
| LKKK**K**SVTV | B*08:01 | A*30 | N | AGERIID**I**IA | B*45:01 | B*53:01 | N |
| LKKK**K**SVTVL | B*08:01 | A*30 | N | GERIID**I**IA | B*45:01 | B*53:01 | N |
| NETPG**I**RYQY | B*18:01 | B*42 | N | GERIID**I**IAT | B*45:01 | B*53:01 | N |
| G**I**RYQYNVL | B*08:01 | B*42 | N | IID**I**IATDI | C*05:01 | B*53:01 | N |
| SPAIFQSSM**T** | B*07:02 | C*16 | N | IQDNS**D**IKV | C*05:01 | B*44 | N |
| AIFQSSM**T**K | A*03:01 | C*16 | N | DNS**D**IKVVPR | A*33:01 | B*44 | N |
| IFQSSM**T**KI | A*23:01 | C*16 | N | NS**D**IKVVPR | A*33:01 | B*44 | N |
| FQSSM**T**KIL | A*02:05 | C*16 | N | AIFQSSM**T**KI | A*03:01 | C*16 | N |
| SM**T**KILEPF | A*23:01 | C*16 | N | RRWRARQR**Q**I | C*06:02 | C*17:01 | N |
| SM**T**KILEPFR | A*31:04 | C*16 | N | A**I**IRILQQL | A*02:05 | A*01 | N |
| M**T**KILEPFR | A*31:04 | C*16 | N | **R**PQVPLRPM | B*07:02 | B*44/C*08 | N |
| HPDKWTVQ**P**I | B*07:02 | A*23 | N | **R**PQVPLRPMT | B*07:02 | B*44/C*08 | N |
| **A**ETFYVDGA | B*45:01 | B*53:01 | N |  |  |  |  |

Supplementary Table 4. Predicted peptides evaluated for HLA adaptation. Amino acid highlighted in red represented the adapted residue. * - Peptide contains and adapted residue to an alternative HLA allele. ** - peptide with amino acid variant M would also be considered HLA adapted

| Seq. In Group | | | Seq. Out Group | | |
| --- | --- | --- | --- | --- | --- |
| Volunteer | Time post EDI | No. Responses | Volunteer | Time post EDI | No. Responses |
| 1 | 2023 | 8 | 13 | 340 | 7 |
| 2 | 336 | 4 | 14 | 339 | 1 |
| 3 | 304 | 21 | 14 | 2518 | 1 |
| 4 | 389 | 30 | 15 | 2210 | 4 |
| 4 | 2208 | 22 | 16 | 341 | 7 |
| 5 | 355 | 5 | 17 | 275 | 6 |
| 5 | 1680 | 6 | 17 | 2205 | 4 |
| 6 | 324 | 1 | 18 | 390 | 3 |
| 6 | 1674 | 1 | 18 | 1736 | 3 |
| 7 | 74 | 3 | 19 | 259 | 5 |
| 7 | 268 | 44 | 19 | 2842 | 11 |
| 8 | 375 | 3 | 20 | 366 | 2 |
| 8 | 2021 | 5 | 20 | 1859 | 5 |
| 9 | 256 | 1 | 21 | 351 | 1 |
| 9 | 1684 | 39 | 22 | 328 | 3 |
| 10 | 351 | 1 | 23 | 330 | 4 |
| 11 | 253 | 9 | 23 | 2358 | 2 |
| 11 | 1508 | 32 | 24 | 339 | 11 |
| 12 | 1840 | 2 | 24 | 2179 | 2 |
|  |  |  | 25 | NA | 2 |
|  |  |  | 26 | NA | 5 |

Supplementary Table 5. ELISPOT responses and days post estimated date of infection of HIV+ volunteers categorized by whether their transmitted founder virus sequence was included within predicted model or excluded from model
